# Bio-Mediated Synthesis and Characterisation of Silver Nanocarrier, and Its Potent Anticancer Action

**DOI:** 10.3390/nano9101423

**Published:** 2019-10-08

**Authors:** Kar Xin Lee, Kamyar Shameli, Shaza Eva Mohamad, Yen Pin Yew, Eleen Dayana Mohamed Isa, Hooi-Yeen Yap, Wei Ling Lim, Sin-Yeang Teow

**Affiliations:** 1 Chemical Energy Conversion and Application (CHeCA), Malaysia-Japan International Institute of Technology (MJIIT), Universiti Teknologi Malaysia, Jalan Sultan Yahya Petra, Kuala Lumpur 54100, Malaysia; hanakarxinlee@gmail.com (K.X.L.); shaza@utm.my (S.E.M.); yenpin_817@hotmail.com (Y.P.Y.); eleen92@gmail.com (E.D.M.I.); 2 Department of Medical Sciences, School of Healthcare and Medical Sciences, Sunway University, Jalan Universiti, Bandar Sunway, Selangor Darul Ehsan 47500, Malaysia; 17084351@imail.sunway.edu.my; 3 Department of Biological Sciences, School of Science and Technology, Sunway University, Jalan Universiti, Bandar Sunway, Selangor Darul Ehsan 47500, Malaysia; weilingl@sunway.edu.my

**Keywords:** green synthesis, silver nanoparticles, nanotechnology, drug delivery, anticancer

## Abstract

Discovery of a potent drug nanocarrier is crucial for cancer therapy in which drugs often face challenges in penetrating efficiently into solid tumours. Here, biosynthesis of silver nanoparticles (AgNPs) using a waste material, *Garcinia mangostana* (GM) fruit peel extract is demonstrated. The best condition for AgNPs synthesis was with 0.5 g of peel extract, 7.5 mM silver nitrate at 45 °C, ~pH 4 for 16 h. The synthesized AgNPs were spherical and 32.7 ± 5.7 nm in size. To test its efficiency to be used as drug carrier, plant-based drug, protocatechuic acid (PCA) was used as a test drug. AgNPs loaded with PCA (AgPCA) resulted in 80% of inhibition at 15.6 µg/mL as compared to AgNPs which only killed 5% of HCT116 colorectal cells at same concentration. The IC_50_ of AgNPs and AgPCA for HCT116 were 40.2 and 10.7 µg/mL, respectively. At 15.6 µg/mL, AgPCA was not toxic to the tested colon normal cells, CCD112. Ag-based drug carrier could also potentially reduce the toxicity of loaded drug as the IC_50_ of PCA alone (148.1 µg/mL) was higher than IC_50_ of AgPCA (10.7 µg/mL) against HCT116. Further, 24-h treatment of 15.6 µg/mL AgPCA resulted in loss of membrane potential in the mitochondria of HCT116 cells and increased level of reaction oxygen species (ROS). These could be the cellular killing mechanisms of AgPCA. Collectively, our findings show the synergistic anticancer activity of AgNPs and PCA, and its potential to be used as a potent anticancer drug nanocarrier.

## 1. Introduction

Nanoparticles (NPs) such as gold (AuNPs), silver (AgNPs), iron (FeNPs), copper (CuNPs), zinc (ZnNPs), platinum (PtNPs) and so on can be synthesized via various methods. Conventionally, NPs synthesis is separated into chemical and physical approaches [1]. In general, chemical approach that uses sodium borohydride, formamide, sodium citrate and ascorbic acid as reducing agents for NPs production is accompanied with toxicity issue [2]. After the synthesis, large amount of surfactant and solvent must be removed before using them for downstream applications [3]. Other alternative such as pro-ecological chemical synthesis has refined the chemical synthesis method by utilising synthetic eco-friendly chemical compounds, hence reducing the use of toxic substances for NP production [4]. On the other hand, physical approach consumes high level of energy which leads to higher cost [3]. Therefore, green synthesis or green method is introduced to overcome these limitations as green synthesis does not involve toxic agents for NP production. The production of NPs using green synthesis is highly simple and facile [5]. NPs can be produced in moderate environment without using elevated temperature or pressure, hence conserving substantial energy which makes it an environmental-friendly and cost-effective method [6]. Among different types of green synthesis method, the most common method is the extracellular NPs production method [4,7]. This method utilises bio-reagents such as bacteria, fungi, enzymes, and plants as both reducing and stabilizing agents [8,9,10]. Amongst all, plants-mediated synthesis is preferred as the morphology of resulting NPs can be easily manipulated by varying different parameters [11,12,13,14]. Each part of plants can be utilised for NPs synthesis, leaves being the most common part as compared to others such as bark, fruit, seed, flower, root and peel [15].

*Garcinia mangostana* (GM) or commonly known as purple mangosteen is a tropical evergreen tree originated from Sunda Islands and the Moluccas of Indonesia. It has an inedible, deep reddish-purple coloured pericarp when becomes ripe. It has been reported that GM has high antioxidant property because the plants extract is rich in phenolic compounds such as xanthones that can be found in pericarp (peels) [16]. In the past, 60 types of xanthones had been isolated from the GM pericarp [17]. Similarly, GM has also been associated with other pharmacological actions such as antitumor, antibacterial, antifungal and antiviral activities [12,13,14,18,19]. Mechanisms of anticancer xanthones have previously been revealed, including induction of apoptosis through regular cell death pathways, suppression of cell proliferation, and inhibition of metastasis by blocking the anti-apoptotic molecules and cell cycle arrest [20].

Silver nanoparticles (AgNPs) have emerged as one of the popular and important NPs for biomedical applications. Recent findings have shown that silver ions and/or AgNPs can be used for wound dressing [21,22], burn treatment [22], antibacterial application [21], dental materials [23], sunscreen lotion [24] and so on. AgNPs possess low toxicity in small quantity, high thermal stability and low volatility [25]. Anticancer property of AgNPs is also being extensively studied. AgNPs is capable to induce the generation of reactive oxygen species (ROS) and thus destroying the mitochondrial respiratory chain of cancer cells. Other cancer killing mechanisms of AgNPs include induction of cell apoptosis and autophagy [26,27], inhibition of cell migration and invasion [27], and induction of lactate dehydrogenase leakage (LDH) [28]. In the past, AgNPs have demonstrated cytotoxic effect towards MCF-7 breast cancer cells at 20 μg/mL for 48 h [25]. AgNPs also suppressed lung cancer cells, H1299 in which 50% of cells were killed at 5 μg/mL [29]. In colorectal cancer cells, AgNPs have shown effective killings at 5 to 28 μg/mL in cell lines such as HCT116 [28], Caco-2 [30] and HT-29 [31]. Furthermore, AgNPs have also been shown to have great potential to act as nanocarrier to deliver anticancer drugs to the cancer cells [32,33]. These evidences highly support the potential use of AgNPs for cancer chemotherapy and chemoprevention in near future. However, concerns regarding the off-target toxicities of AgNPs on the non-cancer or normal cells such as fibroblasts [34], and monocytes [35] have been raised by several research groups before. Hence, it is extremely important to take the specificity of AgNPs into account during the process of AgNPs synthesis and the exploration of its biomedical value.

This study reports the synthesis of AgNPs from GM, and their characterisation using different parameters such as amount of GM peels extract, concentration of AgNO_3_, temperature and pH. The size and shape of AgNPs were determined using UV-visible spectroscopy and transmission electron microscopy (TEM). AgNPs were then loaded with protocatechuic acid (PCA), an anticancer metabolite to investigate its potency as drug nanocarrier. PCA-loaded AgNPs (AgPCA) demonstrated synergistic anticancer effect towards colorectal cancer cell line, HCT116 when compared to the AgNPs and PCA alone. More promisingly, the active dose of AgPCA did not show cytotoxicity towards the colon normal cell line, CCD112 when tested in parallel. To the best of our knowledge, this is the first report showing the potent anticancer action of AgPCA against human colon cancer cells and its possible underlying killing mechanisms.

## 2. Materials and Methods

### 2.1. Materials

GM fruit were collected from an orchard located at Terengganu, Malaysia. Silver nitrate (AgNO_3_, >99.8%) (#19768) was purchased from Acros Organics, UK as a precursor. Sodium hydroxide (#2064), hydrochloric acid (#1137) and phosphate buffer saline (PBS) (#1739) were obtained from R&M Chemicals, UK. Protocatechuic acid (PCA, ≥97%) (#37580) was purchased from Sigma-Aldrich, MO, USA. All reagents used were of analytical grade.

### 2.2. Preparation of Crude GM Fruit Peel Extract

GM peel were collected and washed thoroughly with tap water to remove dirt. The peels were washed again with distilled water before drying in an oven at 40 °C. All the peels were ground into fine powder using an electrical blender and stored in an air tight bottle at room temperature for future use. When needed, fruit peels extracts were prepared using fine powder of GM in 20 mL of deionised water at 60 °C for 30 min.

### 2.3. Synthesis of AgNPs

20 mL of GM extract was mixed with 10 mL of 10 mM AgNO_3_ at 45 °C with continuous stirring for 16 h. Different parameter or condition such as the concentration of plant extract, the concentration of AgNO_3_, temperature and pH were manipulated to determine their effects on the synthesis of AgNPs.

### 2.4. Loading of PCA into AgNPs

10 mL of nanoparticle suspension was added to 50 mL of 5 mg/mL PCA aqueous solution. The reaction mixture was left to occur with ultra-sonication (30 amplitudes) for 1 h. The obtained drug loaded AgNPs (AgPCA) was centrifuged and washed repeatedly for 3 times before drying in the oven for further characterisation.

### 2.5. Characterisation

UV-2600 UV-Vis Spectrophotometer (Shimadzu, MD, USA) was used to determine the formation of NPs in the range of 220 to 800 nm. Peaks at the area 400–500 nm indicate the formation of AgNPs. After UV-visible spectroscopy, all of the samples were dried in powder form and collected for further characterisation. Transmission electron microscopy (TEM) images were recorded using JEM-2100F high-resolution transmission electron microscope (HR-TEM) from JEOL, Tokyo, Japan to determine the morphology, size and distribution of the samples. Crystallinity of the NPs was confirmed using Empyrean X-ray diffractometer (XRD) from Malvern Panalytical, UK where the powder samples were analysed at 45 kV and 40 mA over a range of 2θ = 30–80°. Infrared spectroscopy (IRTracer-100, Shimadzu, MD, USA) was used to detect the functional groups present in the synthesized NPs. Spectra were recorded with 16 scan per sample at the range of 600–4000 cm^−1^. STA 449 F3 Jupiter (NETZSCH, Selb, Germany) was used to conduct thermogravimetric analysis (TGA) for the drug loaded AgNPs at a heating rate of 10 °C/min under nitrogen flow at a rate of 50mL/min. Encapsulation efficiency was calculated based on the TGA analysis. Encapsulation efficiency is the amount of drug loaded per unit weight of the NPs [36]. The drug encapsulation efficiency was calculated using the equation below:

(1) $$\mathrm{Encapsulation}\;\mathrm{efficiency}\;=\;\frac{\left(\mathrm{total}\;\mathrm{drug}-\mathrm{free}\;\mathrm{drug}\right)}{\mathrm{total}\;\mathrm{drug}}\;\times \;100\;[\%]$$

### 2.6. Drug Release

5 mg AgNPs were suspended in 5 mL PBS (adjusted to pH 5.0) which was then placed into dialysis bags with the two ends tied. The dialysis bags were immersed fully into 40 mL of the release medium (PBS) with constant stirring of 100 rpm. The whole system was incubated at 37 °C. 1 mL aliquot was sampled from the system at 15-min interval for the first hour and every hour afterwards for 25 h. An equal volume (1 mL) of fresh medium was immediately replenished to keep the volume constant. The collected samples were then analysed using UV-visible spectrophotometer at 250 nm.

### 2.7. Cell Culture

HCT116 (ATCC CCL-247) colorectal carcinoma and CCD112 (ATCC CRL-1541) colon normal cell lines were purchased from American Type Culture Collection (ATCC, VA, USA). Both cell lines were maintained in Dulbecco’s Modified Eagle’s medium (DMEM, #12800) supplemented with 10% fetal bovine serum (FBS) (#16000-044) and 1% penicillin/ streptomycin (#15140122) from Life Technologies, CA, USA. CellTiter 96 Aqueous One Solution or MTS reagent (#G3582, Promega, WI, USA) was purchased to evaluate the cytotoxicity of NPs on the cell lines as described below.

### 2.8. Cytotoxicity Assay

To determine the cellular killing effect of NPs, MTS assay was performed according to the manufacturer’s instruction with slight modification as previously described by our group [13]. Briefly, 5000 HCT116 and CCD112 cells per well (100 µL/well) were seeded onto a 96-well plate and incubated overnight at 37 °C in a 5% CO_2_ incubator. The next day, 2-fold serially diluted NPs (250, 125, 62.5, 31.3, 15.6, 7.8, 3.9, 0 µg/mL) (100 µL/well) were added into the wells and the plate was incubated for 72 h at 37 °C in the 5% CO_2_ incubator. Then 20 µL MTS reagent per well was added into the plate and incubated for additional 3 h at 37 °C in the 5% CO_2_ incubator. The optical density (OD) was then measured at 490 nm using a multimode microplate reader (Tecan, Switzerland). The dose response graph was plotted by calculating the percent cell viability using the formula below:

(2) $$\%\mathrm{Cell}\;\mathrm{viability}\;=\;\frac{\mathrm{OD}\;\mathrm{of}\;\mathrm{sample}\;\mathrm{well}\;(\mathrm{mean})}{\mathrm{OD}\;\mathrm{of}\;\mathrm{control}\;\mathrm{well}\;(\mathrm{mean})}\;\times \;100$$

In addition, the inhibitory concentration causing 50% growth inhibition (IC_50_ value) was also determined using an online calculator (https://www.aatbio.com/tools/ic50-calculator). The images of cells treated with/ without the NPs were captured using an inverted microscope attached to a camera system (IM3 Phase contrast, Optika, Italy) at magnification of 10×.

### 2.9. Mitochondrial Membrane Potential (JC-1) Assay

Cell vitality status was evaluated by examining the cellular mitochondrial function using JC-1 Mitochondrial Membrane Potential Assay Kit (#10009172, Cayman Chemical, MI, USA) following the manufacturer’s instruction. HCT116 cells were treated with 15.6 µg/mL AgPCA for 24 h as previously described [28,30] and the mitochondrial behaviour was assessed through JC-1 staining. In healthy cells, JC-1 forms complexes and form aggregates in red fluorescence meanwhile unhealthy cells (i.e., cells undergoing apoptosis) exhibit green fluorescence. The health status of cells was measured using microplate reader method and fluorescence microscopy. Samples were observed under an inverted fluorescence microscope (Nikon Eclipse Ti-S, Japan) and images were captured using the Nikon NIS-Elements (Japan) microscope imaging software.

### 2.10. Reactive Oxygen/Superoxide Species Assay

Real-time ROS and superoxide production in cells was measured by flow cytometry (BD FACSCalibur, NJ, USA) using ROS-ID Total ROS/Superoxide detection kit (#ENZ-51010, Enzo Life Sciences, NY, USA). 2 × 10^5^ HCT116 cells/well were seeded onto a 12-well plate, treated with 15.6 µg/mL AgPCA for 24 h as previously described [28,30] and processed following the manufacturer’s instruction. Cells treated with 200 µM ROS inducer, pyocyanin and 5 mM ROS inhibitor, N-acetyl-L-cysteine (provided by the kit) were used as the positive and negative controls, respectively. Data was analysed and dot plots was plotted using CellQuest Pro software (BD, NJ, USA). To reconfirm the data, fluorescence microplate method was performed on a black 96-well plate (Pelkin Elmer ViewPlate) using the same kit. After the cells treatment as abovementioned, cells were processed following the manufacturer’s instruction. Fluorescence intensity was measured by microplate reader at fluorescein (Ex/Em = 488/520 nm) and rhodamine (Ex/Em = 550/610 nm) representative of ROS and superoxide levels, respectively.

### 2.11. Statistical Analysis

Independent experiments were repeated at least three times, and the data were expressed as mean ± standard deviation for all triplicates within an individual experiment. Data were analysed using a Student’s *t* test *p* < 0.05 was considered significant.

## 3. Results

**Figure 1:**
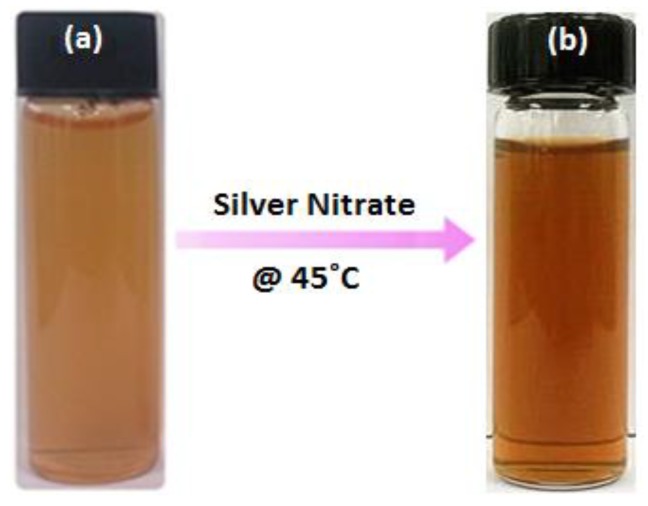
Change of colour after the addition of AgNO_3_**.** (**a**) crude GM peels extract and (**b**) crude GM peels extract with addition of AgNPs solution.

AgNPs were synthesized using GM crude peels extract which act as reducing and stabilising agent while AgNO_3_ (silver nitrate) solution acted as Ag precursor. The reaction temperature was maintained at 45 °C with continuous stirring at 500 rpm. The reduction of AgNO_3_ was indicated by the colour change from light brown to dark brown after the addition of AgNO_3_ as shown in Figure 1.

### 3.1. UV-Visible (UV-Vis) Spectroscopy

**Figure 2:**
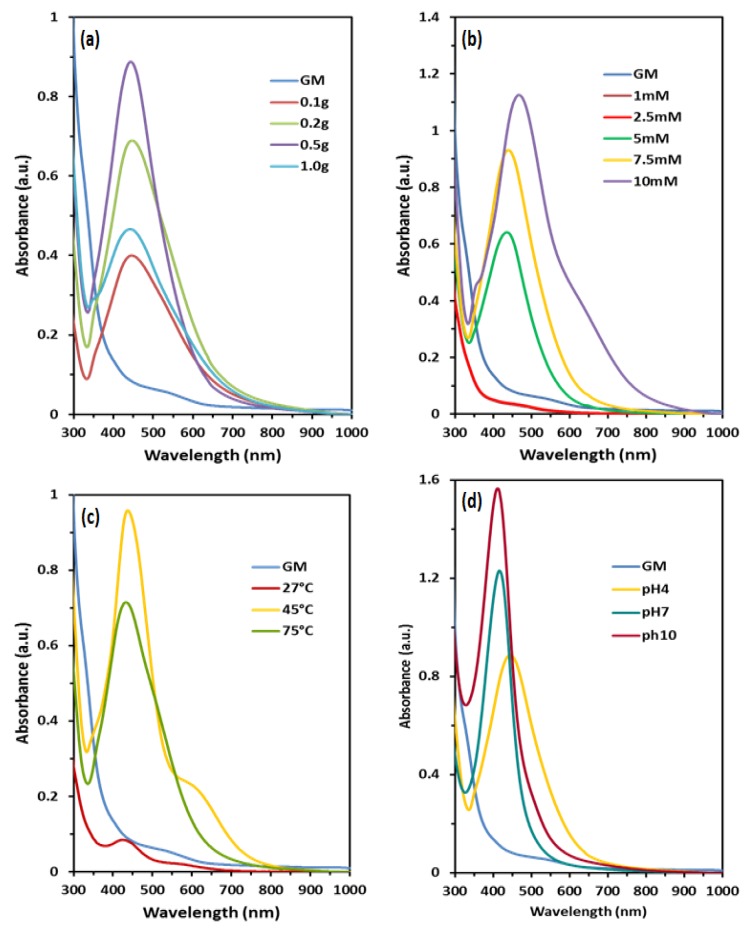
UV-visible spectra of AgNPs synthesized with various parameters: (**a**) amount of peels powder, (**b**) concentration of AgNO_3_, (**c**) temperature and (**d**) pH.

The effect of the amount of GM peels powder on the formation of AgNPs was evaluated using UV-vis. As shown in Figure 2a, the UV peaks become sharper following the increase of peels powder. 0.5 g of GM peels powder showed the sharpest peak. However, intensity of 1.0 g peak dropped and a broad peak was obtained. The spectrum of 0.5 g of GM peels powder reacting with 10 mM AgNO_3_ gave the most intense peak at the position of 443 nm. Concentration-dependent shift of peaks was observed following the increased concentration of AgNO_3_ (Figure 2b). 1 mM and 2.5 mM did not contribute to the production of AgNPs because no increase in intensity were observed. When the concentration of AgNO_3_ increased, the intensity of UV-vis peak absorbance increased. 10 mM of AgNO_3_ gave the most intense peak at 465 nm. Red shift was observed from 433 nm (5 mM) to 465 nm (10 mM), suggesting that aggregates or larger size of NPs were formed [37]. From Figure 2c, the reaction temperature of 45 °C gave the highest peak at 437 nm. A blue shift was observed from 439 nm (27 °C) to 434 nm (75 °C). Subsequently, the effect of pH was investigated using NaOH. As shown in Figure 2d, rapid reaction was observed when the pH of the GM crude extract turned into alkaline. Absorbance peak was observed at 443 nm, 414 nm and 410 nm when the pH of the GM extract increased to pH 4, 7 and 10, respectively.

### 3.2. Transmission Electron Microscopy (TEM)

**Figure 3:**
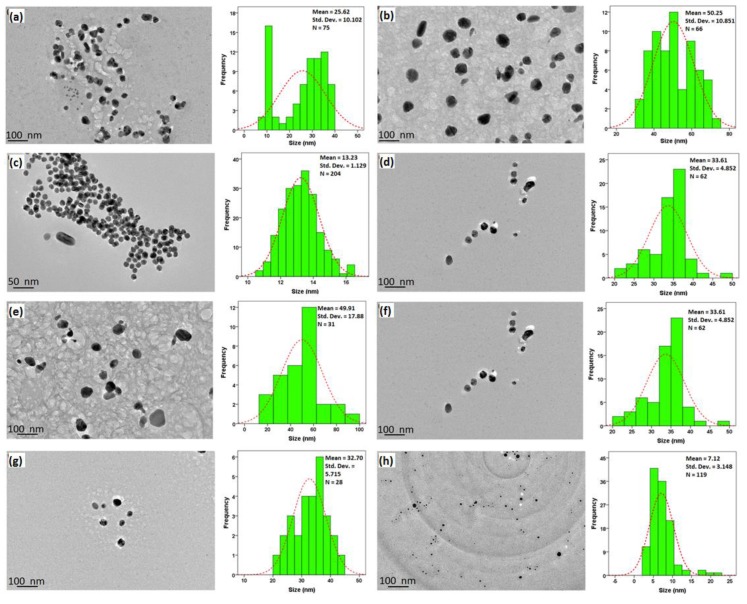
TEM of AgNPs synthesized by various parameters: amount of peel powder, (**a**) 0.2 g, (**b**) 0.5 g; concentration of AgNO_3_, (**c**) 2 mM, (**d**) 7.5 mM; temperature: (**e**) 27 °C, (**f**) 45 °C; and pH (**g**) pH 4 (**h**) pH 7.

The size and shape of AgNPs were examined using TEM. As shown in Figure 3a,b the amount of peels powder significantly affected the formation of AgNPs. With 0.2 g of GM peels powder, AgNPs had wider particles size distribution. The shape of AgNPs was mostly spherical with some rod shape. At 0.5 g GM powder, the size of AgNPs increased from average size of 25.62 to 50.25 nm. Figure 3c,d show the AgNPs synthesized at 2 and 7 mM AgNO_3_. At 2 mM, the mean size of NPs was 13.23 nm. As shown in Figure 3c, the smaller NPs tend to aggregate together while bigger size NPs did not show the same observation. Low concentration (2 mM) of AgNO_3_ produced smaller NPs as compared to the higher concentration (7.5 mM). However, 7.5 mM of AgNO_3_ produced AgNPs with better dispersion. The effect of reaction temperature was also studied as shown in Figure 3e,f. At room temperature (27 °C), distorted spherical shape NPs with an average size of 49.91 nm were observed. At moderate temperature (45 °C), more uniform spherical AgNPs with an average size of 33.61 nm were produced. Lastly, the effect of pH was also studied as shown in Figure 3g,h. The mean size of AgNPs at pH 4 was 32.7 nm while most of the NPs were spherical in shape. When the pH of the extract environment increased to pH 7, the average size of the NPs decreased to 7.12 nm. The AgNPs were well-distributed as shown in Figure 3h. Notably, the samples became unstable and degraded at pH 7 during the TEM preparation.

### 3.3. X-ray Diffraction (XRD) Analysis

**Figure 4:**
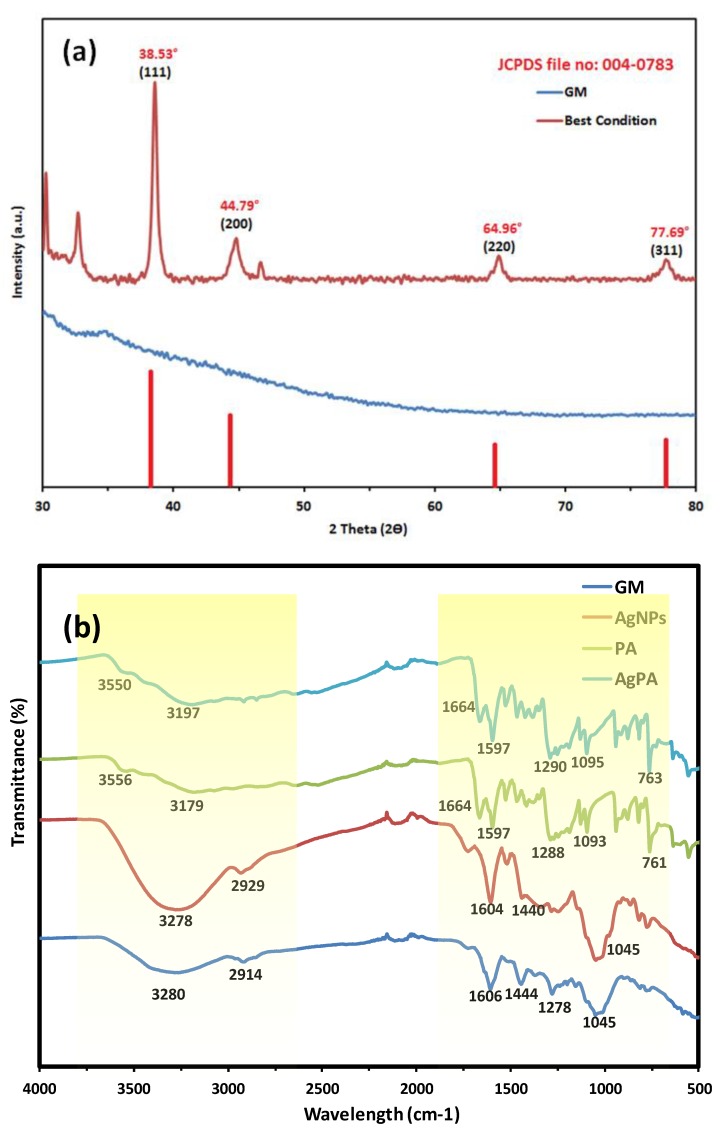
(**a**) XRD spectra of AgNPs and the intensity peak of reference data and (**b**) FTIR graphs of GM, AgNPs, PCA and AgPCA.

AgNPs were synthesized using the optimal conditions (0.5 g peels powder, 7.5 mM AgNO_3_, 45 °C, pH 4) and analysed by XRD as shown in Figure 4a. Intense peaks were observed at 2θ = 38.53°, 44.79°, 64.96° and 77.69° which corresponded to the (111), (200), (220) and (311) planes, representing the fcc structure of metallic Ag. Three additional peaks were observed in the XRD pattern at 30.19°, 32.74° and 46.76°. The particle size of AgNPs can be estimated using the the Debye-Scherrer equation. Using the Scherrer equation, 26 nm was calculated to be the average crystallite size of the AgNPs.

### 3.4. Fourier-Transform Infrared Spectroscopy (FT-IR)

As shown in Figure 4b, major stretching appeared at the region of 3000–3500 cm^−1^ and 1000–1600 cm^−1^ for both GM and AgNPs spectra. After the loading of PCA, the spectrum of AgPCA changed and become similar to the spectrum of PCA. Both GM and PCA contains O-H stretch in their structure originally at region of 3280 cm^−1^ and 3179 cm^−1^ respectively as shown in Figure 4b. The presence of O-H stretch at 3278 cm^−1^ and 3280 cm^−1^ for AgNPs and GM respectively, are related to phenols and flavonoids [38]. During the synthesis of AgNPs, the shifting of O-H stretch from 3280 cm^−1^ to 3278 cm^−1^ is related to the binding of AgNPs to the GM extract [39]. After the loading of PCA onto the AgNPs, shifting occured from 3179 cm^−1^ to 3197 cm^−1^, suggesting that reaction occured at this stretch.

The presence of C-H bond was detected at the region of 2914 cm^−1^ (GM) and 2929 cm^−1^ (AgNPs), suggesting that xanthone and other compounds in the peels extract were present [40]. The region of 1700 cm^−1^ shows the presence of C = O stretching for both GM and AgNPs [41]. The increase of intensity indicated that more C = O bonds were formed following the initiation of reaction.

C-C ring aromatic bond was shown at the region of 1500–1600 cm^−1^. This confirmed the presence of aromatics structure in the GM extract. Another C-C aromatics stretch was observed in both spectra at the region of 1440 cm^−1^ and 1444 cm^−1^ for AgNPs and GM, respectively. This data is highly associated with the aromatic backbone of organic compounds that can be found mainly in the pericarp of GM [42].

**Figure 5:**
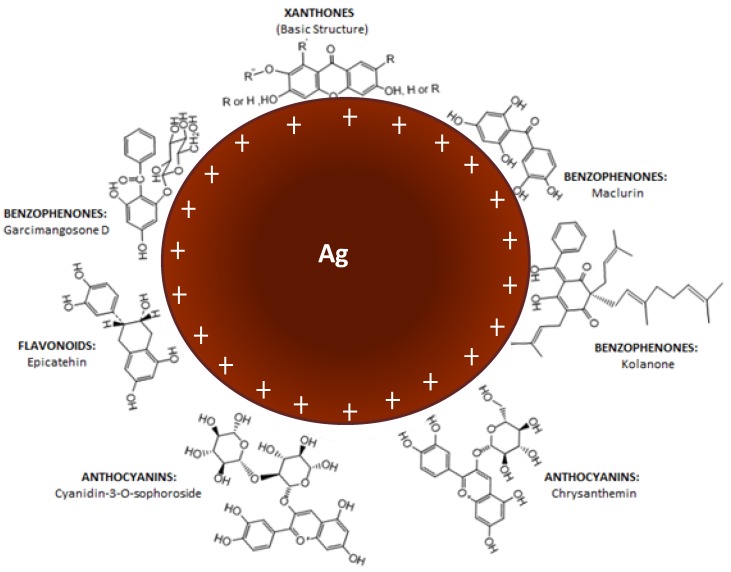
Simple schematic illustration of interaction between AgNPs and the active functional groups present in the GM peels extract.

Finally, the band at 1045 cm^−1^ for both spectra was associated to C-O-C stretch [41]. All the above results match with xanthone, flavonoids, and other compounds that could derived from the pericarp of GM [18,43]. A simple schematic illustration is based on the known organic compounds present in GM peels extract was presented as shown in Figure 5.

### 3.5. Thermogravimetric Analysis (TGA)

**Figure 6:**
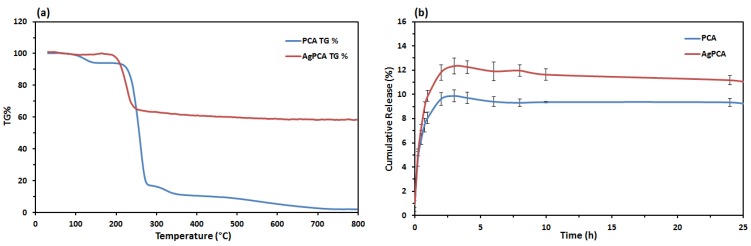
Thermogravimetric analysis and drug profile release of PCA and Ag-PCA. (**a**) TGA thermograms of PCA and AgPCA; (**b**) Drug release profile of PCA and AgPCA.

After sonication of AgNPs with PCA for 5 h, the loading was confirmed using UV-vis spectroscopy as shown in Figure S1 where two peaks (250 nm and 288 nm) related to PCA were observed. AgPCA was then analysed using TGA to calculate the loading efficiency of AgNPs because TGA can confirms the content of the PCA and PCA loaded AgNPs as shown in Figure 6a [44]. Figure 5a indicated the thermal analysis of the drug and drug-loaded AgNPs. PCA showed three main weight loss stages. The first weight loss stage (4.64%) occurred at 138 °C. Second stage of weight loss (77.64%) occurred at 200–299 °C. The last stage of weight loss (6%) occurred at the region of 299–350 °C. Drug-loaded NPs only showed single stage of weight loss at the region of 240–299 °C. From the TGA data, encapsulation efficiency and drug loading efficiency were calculated. The encapsulation efficiency of AgNPs was 38.05%.

### 3.6. Drug Release Study

In Figure 6b, PCA solution that acts as a control had a rapid release in about 3 h with a cumulative release of 9.7%. The release rate of AgPCA was faster as compared to the PCA and the release process completed in about 4–5 h. A release profile of the first hour was demonstrated in Figure S2 to show the release rate more clearly in the early stage. The cumulative release percent of AgPCA was 12%. Release of the samples achieved equilibrium after 12 h and no further changes occurred at 25 h. A drop was observed at the 4th hours because the release of PCA slowed down at this point. The system was diluted when the fresh medium was added after the sampling process [45]. Based on the drug release profile, burst release of AgPCA occurred in the first 15 min. After that, the remaining PCA were released in a sustained manner for about 6 h before achieving equilibrium. These drug release data were fitted into five mathematical models (zero order, first order, Hixon-Crowell, Higuchi and Korsmeyer-Peppas) to understand the mechanism and kinetics of the drug delivery system. AgPCA fitted into the Higuchi method with R^2^ of 0.9775 and Korsmeyer-Peppas model with R^2^ of 0.9710 (Table 1).

### 3.7. In Vitro Cytotoxicity Assay

**Figure 7:**
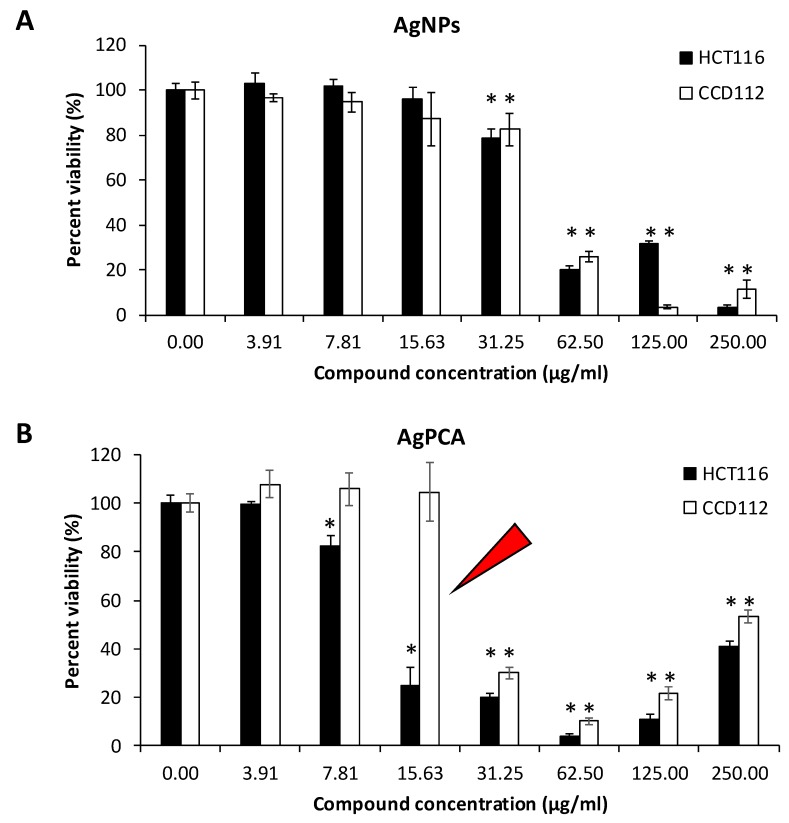
Cytotoxic effects of nanoparticles (**A**) AgNPs and (**B**) AgPCA against colorectal cancer (HCT116) and colon normal (CCD112) cells. Red triangle shows the selectivity activity of AgPCA on HCT116, but not CCD112. Data are expressed as mean + standard deviation for triplicates within an individual experiment. Statistical significance was performed using Student’s *t* test. (* *p* < 0.05).

**Figure 8:**
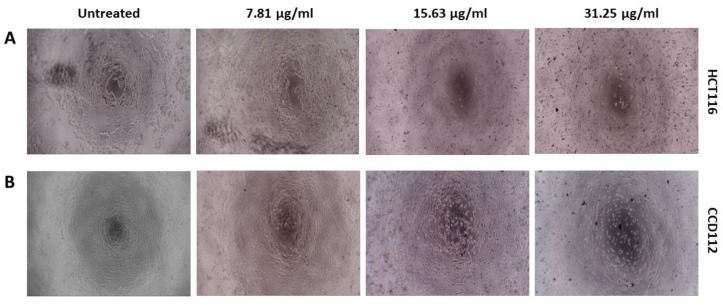
Microscopic examination of (**A**) HCT116 and (**B**) CCD112 cells treated with 7.81, 15.63 and 31.25 µg/mL of AgPCA. Images were captured at magnification of 10X.

Cytotoxic effect of PCA, AgNPs and AgPCA were determined on colon-derived HCT116 (cancerous) and CCD112 (normal) cells up to 250 µg/mL. Table 2 shows the calculated IC_50_ values for all the tested compounds. Both AgNPs and AgPCA showed cytotoxic effects against HCT116 cells. Based on the calculated IC_50_, the AgPCA (IC_50_ = 10.73 µg/mL) was more effective than the AgNPs (IC_50_ = 40.23 µg/mL). The results also showed that AgPCA was more potent against HCT116 than the normal cells (IC_50_ = 27.4 µg/mL). As shown in Figure 7, dose-dependent inhibitions were observed. 31.3 µg/mL and higher concentrations of AgNPs significantly killed both HCT116 and CCD112 cells (*p* < 0.05). The level of cytotoxic effect in HCT116 and CCD112 did not show any difference in AgNPs in most of the tested concentrations (Figure 7A). In contrast, several tested concentrations of AgPCA (7.81, 15.6, and 31.25 µg/mL) showed higher cytotoxic effect in HCT116 compared to CCD112. Notably, 15.6 µg/mL AgPCA killed almost 80% of HCT116 while did not affect the CCD112 cells (shown by a red triangle in Figure 7B). This was further supported by microscopic examination which showed high percentage of cell killing in HCT116 but not in CCD112 cells when tested at 15.6 µg/mL (Figure 8).

### 3.8. Mitochondrial Membrane Potential Assay

**Figure 9:**
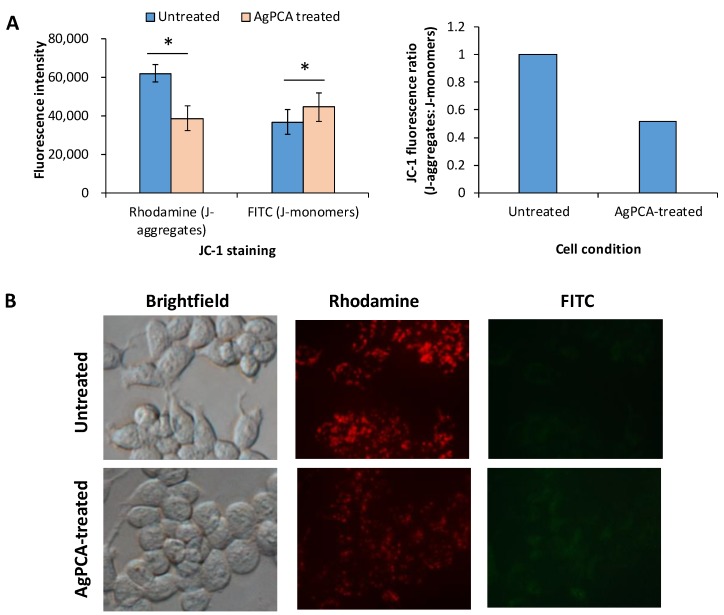
Mitochondrial membrane potential analysis of untreated control and AgPCA-treated HCT116 cells. (**A**) Fluorescence intensity (left) and fluorescence ratio of J-aggregates (rhodamine) to J-monomers (FITC) (right). Data are expressed as mean + standard deviation for triplicates within an individual experiment. Statistical significance was performed using Student’s *t* test. (* *p* < 0.05). (**B**) Fluorescence microscopy of JC-1 staining. FITC (diffuse green J-monomers) indicates the unhealthy status while rhodamine (red mitochondrial J-aggregates) indicates the healthy status. Images were captured at magnification of 40X.

In addition to MTS assay, the mitochondrial function of AgPCA-treated HCT116 cells was examined using a commercial kit which is another assay for cell vitality/health determination. Since 15.6 µg/mL of AgPCA killed almost 80% of HCT116 while remain non-toxic to the normal cells, this concentration was used for this experiment. In this assay, a cytofluorimetric cationic dye, JC-1 forms complexes known as J-aggregates with red fluorescence (rhodamine) in healthy cells, while JC-1 remains in monomeric form and exhibits green fluorescence (FITC) in unhealthy cells. As shown in Figure 9A, AgPCA-treated cells showed significantly lower red fluorescence (healthy signal) and higher green fluorescence (unhealthy signal) respectively when compared to the untreated control (*p* < 0.05). The calculated ratio of J-aggregates to J-monomers for the AgPCA-treated cells gave 0.52, indicating the higher number of unhealthy cells. This result is also supported by fluorescence microscopy in which AgPCA-treated cells showed dimmer red J-aggregates and slightly brighter green J-monomers compared to the untreated control at a normalised background (Figure 9B).

### 3.9. Oxidative Stress and Superoxide Assay

**Figure 10:**
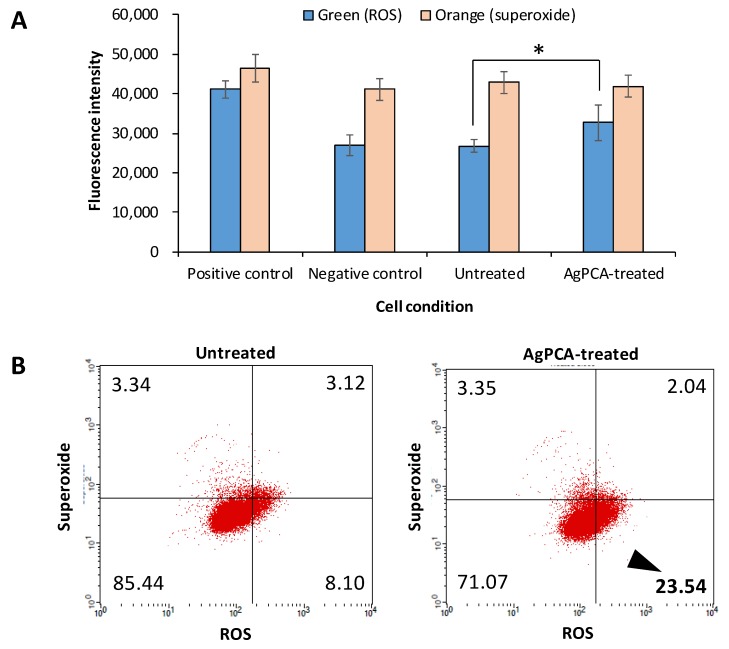
Reaction oxygen species (ROS) and superoxide production of untreated control and AgPCA-treated HCT116 cells. (**A**) Fluorescence intensity of green (ROS) and orange (superoxide). Data are expressed as mean + standard deviation for triplicates within an individual experiment. Statistical significance was performed using Student’s *t* test. (* *p* < 0.05). (**B**) Dot-plots by flow cytometry. Untreated cells were used as control. Cell debris were ungated. The numbers reflect the percentage of the cells in each quadrant and are represented by average of three independent experiments. Black triangle shows the increased level of ROS in AgPCA-treated cells.

Oxidative stress and superoxide were measured using a commercial assay kit. The green and orange fluorescence probes detect the ROS and superoxide, respectively. As shown in Figure 10A, the increase of ROS and superoxide production were evident in the pyocanin-treated positive control. Compared to the untreated control, the AgPCA-treated HCT116 cells generated significantly higher level of ROS (*p* < 0.05) but not in the superoxide production. This was further supported by the flow cytometry which showed the increase of ROS from 8.1 to 23.54% (about 3-fold) after 24 h exposure of AgPCA (Figure 10B).

## 4. Discussion

GM is rich in phenolic compounds such as xanthones, α-mangostana, β-mangostana and flavonoids [17]. Abundant hydroxyl groups were available in these compounds for the reduction of AgNPs [46]. It can be speculated that the bioreduction of Ag was through the oxidation of hydroxyl group which can be represented as [46,47]:

Ag^+^ + R-OH → Ag^0^ + R=O + H^+^ (3)

Based on the UV-vis results, an increased in the amount of GM peels powder and concentration of AgNO_3_ increased the peak intensity of UV-vis spectra. This result suggests that higher amount of GM peels powder and concentration of AgNO_3_ speed up the formation of AgNPs as more AgNPs were formed which is consistent with the previous result [48]. Red shift was observed due to the increase of interactions between GM peels extract and available silver ions, hence resulting in the increase of AgNPs size [49]. GM at 1.0 g spectrum showed a reduction in absorption band because of the aggregation of AgNPs causing the depletion of stable NPs [50]. From our data, 0.5 g of GM peels powder appears to be the best condition for the synthesis of AgNPs because it gave an intense peek at 443 nm. Meanwhile, 7.5 mM of AgNO_3_ appears to be the best condition because it gave a sharp peak with minimal shift from 433 nm to 441 nm. Furthermore, smaller NPs was formed following the increase in temperature from 27 °C to 75 °C as a blue shift was observed. This result suggests that the increase in temperature leads to the rapid consumption of reactants to form the nuclei and generate small NPs rather than aggregates [51]. The peak position of AgNPs in acidic condition was at a higher wavelength, suggesting that the aggregation occurred which might be due to the anion electrostatic repulsion in the solution [37]. The reduction of AgNPs at different pH conditions is believed to be caused by the variation of functional groups in the GM peels extract [51]. Broader tailed absorption bands around 550–700 nm were observed for 10 mM and 45 °C because of the different modes of plasmon excitation from the anisotropic morphology AgNPs [52].

TEM results suggested that the size of the AgNPs increased with an increased amount of GM peels powder. This result is consistent with the previously reported work [40]. From the observation, low quantity of GM extract cannot prevent aggregation of the NPs, it was only sufficient for the reduction of Ag precursor. In contrast, high quantity of GM extract caused excessive growth of the NPs which is in good agreement with the UV-vis result in which a red shift was observed [53]. Increase in the concentration AgNO_3_ produced monodispersed AgNPs that were bigger in size because nucleation was favoured at low AgNO_3_ concentration, thus forming AgNPs with smaller size. When the concentration of AgNO_3_ increased, NPs growth dominated the reduction process, thus produceing larger AgNPs [54]. In addition, the reaction temperature significantly influenced the particle size of AgNPs. Smaller size of NPs was produced at a higher temperature, possibly due to the homogenuos nucleation and rapid reduction of AgNO_3_ at high temperature [38]. Smaller AgNPs were produced in pH 7 which is constent with a previous work [54]. This result is in accordance with the UV-vis result in which a blue shift was observed following the increase of pH. This finding suggests that pH also plays crucial role in the production of AgNPs. Overall, the TEM images suggest that the optimal parameters to achieve desirable size and shape of the AgNPs for drug loading are to use 0.5 g GM peels powder with 7.5 mM AgNO_3_ and stirred for 16 h at 45 °C and pH 4.

The XRD result is in line with the retrived JCPDS file no. 00-004-0783 database [55]. Another three additional peaks were corresponded to the unreacted AgNO_3_ that matched the JCPDS file no. 00-006-0363. These peaks might also be caused by other phytochemical compounds in the extract [42]. AgNPs produced were fcc in nature.

FTIR results suggested that the presence of O-H stretch were related to phenols and flavonoids [38]. The shifting of O-H stretch were related to the binding of AgNPs to the GM extract and PCA to AgNPs [39,56]. The present of C-C ring aromatic bond at the region of 1600–1500 cm^−1^ suggests that aromatics structure exists in both the GM extract and PCA. C-C aromatic stretchs were observed in the spectra of GM and AgNPs only. It was relevant to the aromatic back bone of organic compounds that can be found mainly in the pericarp of GM [42]. All the above results matched with the xanthone, flavonoids, and other compounds that can be derived from the pericarp of GM [18,43]. Slight shifting of the C = O bond was observed for both spectra of PCA and AgPCA. This was most probably due to the occurance of hydrogen bonding [56]. Reduction in intensity at 941 cm^−1^ is related to the hydroxyl moiety of carboxylic group. This result suggests that the reaction occured between the –COOH groups of PCA and AgNPs [57]. In addition, the FTIR analysis suggested that O-H groups were closely associated in the stabilization of AgNPs. Surface of the AgNPs is speculated to be positively charged so that the negatively charged O-H groups can take part in the stabilisation process (Figure 5).

Thermal analysis was carried out to analyse the drug-loaded AgNPs. PCA gave 3 stages of weight lost where the first stage was corresponded to the loss of absorbed water at 138 °C as shown in the previous work [57]. Second stage of weight lost was related to the decomposition combustion of PCA [58]. Finally, the last stage of weight lost was due to the decomposition of PCA residue [59]. AgPCA only showed one stage of weight lost which was due to the combustion of PCA. The drug-loaded NPs appear to be more thermally stable as compared to PCA.

From the drug release profile, burst released occur at the first 15 min indicating that some PCA is weakly bound or adsorbed on the large surface area of the NPs [57,60]. After that, PCA that was loaded on the NPs was released in a stable manner. Kinetics assesments suggest that the drug were released through both diffusion and erosion controlled (non-fickian) method because the release data can be fitted into Higuchi and Korsmeyer-Peppas models [61]. Dissolution-filling approach occurred as PCA gradually released and diffused from the NPs that act as the carriers [62].

The IC_50_ result suggests that combining AgNPs with PCA was beneficial in killing the HCT116 cells. There is an improvement on the anticancer effect of AgPCA as compared to AgNPs and PCA alone. Moreover, the loaded NPs showed a better selectivity on HCT116 cells. This result suggests that AgNPs can be an effective nanocarrier for anticancer drug delivery. In the attempt to determine the killing mechanism, we showed that AgPCA treatment impaired the mitochondrial function and increased the ROS generation in HCT116 cells, which is in line with the previous finding [28]. These physiological changes could be due to the leaching of Ag ions and the release of PCA from AgPCA which eventually induce apoptosis or necrosis in the treated cells [28,30]. ROS-mediated oxidative stress plays important regulatory role in cellular homeostasis. When the process is interfered by the Ag ions or PCA, cell death occurs due to the imbalance between pro- and anti-oxidants levels [33].

This study highlights the great potential of AgNPs to be used as a non-toxic anticancer drug delivery system. Our future works will focus on the control of the physiochemical properties of AgNPs to suit its use for future cancer treatment. One of the strengths in nanotechnology is the capacity to modify the NPs’ physiochemical characteristics such as size, charge, electrical properties, shape, viscosity, and surface tension that have direct impact on their biological moiety, cytotoxicity, and stability. In addition to the optimisation of NPs’ modifications, formulation and synthesis for cancer treatment, there are underlying challenges to adopt NPs for clinic use [32,33]. Some of the key challenges to address are their activity in the more stringent biological systems such as animal models, administration method, controlled release and targeted delivery, possible resistance developed by tumour, and cost-effectiveness from industrial and pharmaceutical perspectives [32,33].

## 5. Conclusions

This study proposes an environmental friendly method via the usage of *Garcinia mangostana* fruit peels extract for the synthesis of AgNPs. This method fully utilises unwanted waste material to a better use. The size and shape of the AgNPs can be easily controlled by manipulating various parameters as reported in this study. Our results showed that the generated AgNPs are suitable to act as nanocarriers in which the PCA could be loaded easily. The anticancer assay confirmed that the PCA-loaded AgNPs possess a better anticancer property as compared to the NPs alone while reducing the toxicity of loaded drug. Some of the cell death mechanisms of AgPCA on HCT116 cells include mitochondria-related apoptosis and ROS generation. Overall, using PCA as an example, the results suggest that the biogenic AgNPs have high potential to be utilised as an anticancer drug delivery system.

## Figures and Tables

**Table 1:** Kinetic assesment of AgPCA based on various kinetic mathematical models.

Sample	Correlation Coefficient of Model (R^2^)				
	Zero Order	First Order	Hixon-Crowell	Higuchi	Korsmeyer-Peppas
AgPCA	0.7918	0.7712	0.7651	0.9775	0.9710

**Table 2:** IC_50_ values of protocatechuic acid (PCA), silver nanoparticles (AgNPs) and PCA loaded AgNPs (AgPCA) against two cell lines.

Cell Line	IC_50_ with Standard Deviation (µg/mL)		
	PCA	AgNPs	AgPCA
HCT116 (cancerous)	148.1 ± 3.72	40.2 ± 2.28	10.7 ± 3.79
CCD112 (normal)	224.4 ± 3.77	47.0 ± 6.59	27.4 ± 1.50

## Supplementary Materials

The following are available online at https://www.mdpi.com/2079-4991/9/10/1423/s1, Figure S1: A comparison of UV-vis spectrum between PCA and AgPCA. Figure S2: Drug release profile of PCA and AgPCA at the first hour.
