# Supplementary figures and images for: Bio-Mediated Synthesis and Characterisation of Silver Nanocarrier, and Its Potent Anticancer Action
Source: Nanomaterials (Basel). 2019 Oct 8;9(10):1423. doi: 10.3390/nano9101423 (PMC6835987; doi:10.3390/nano9101423)

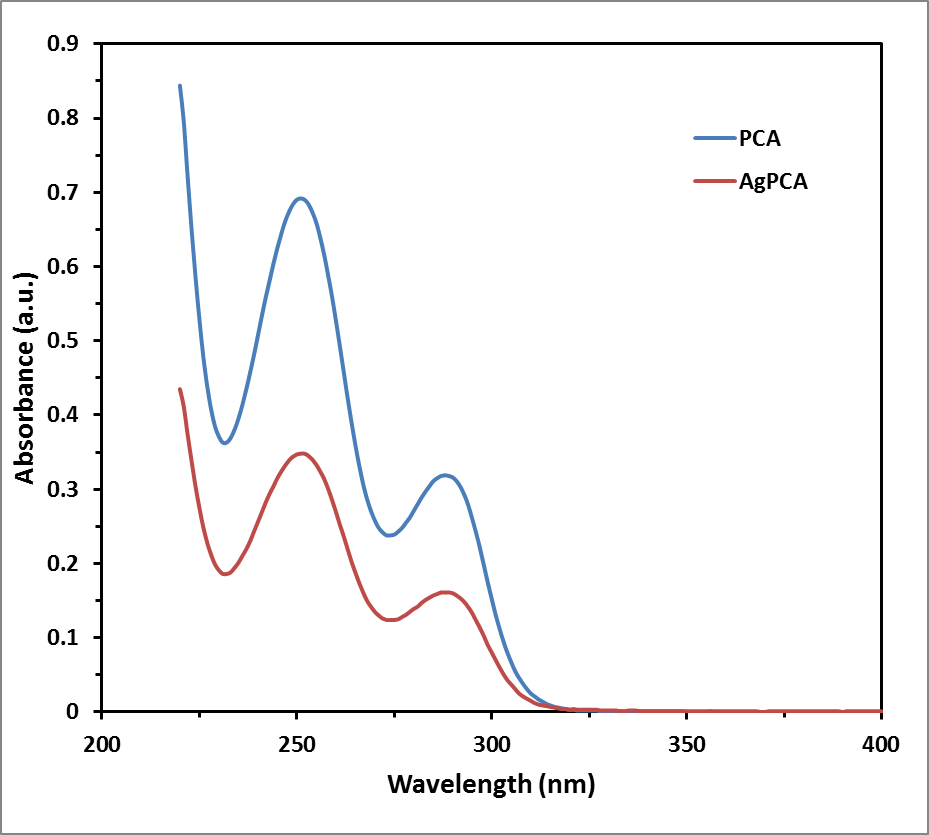

Supplement: Supplementary file 1 [file nanomaterials-09-01423-s001.zip › Supplement/Figure S1.docx]

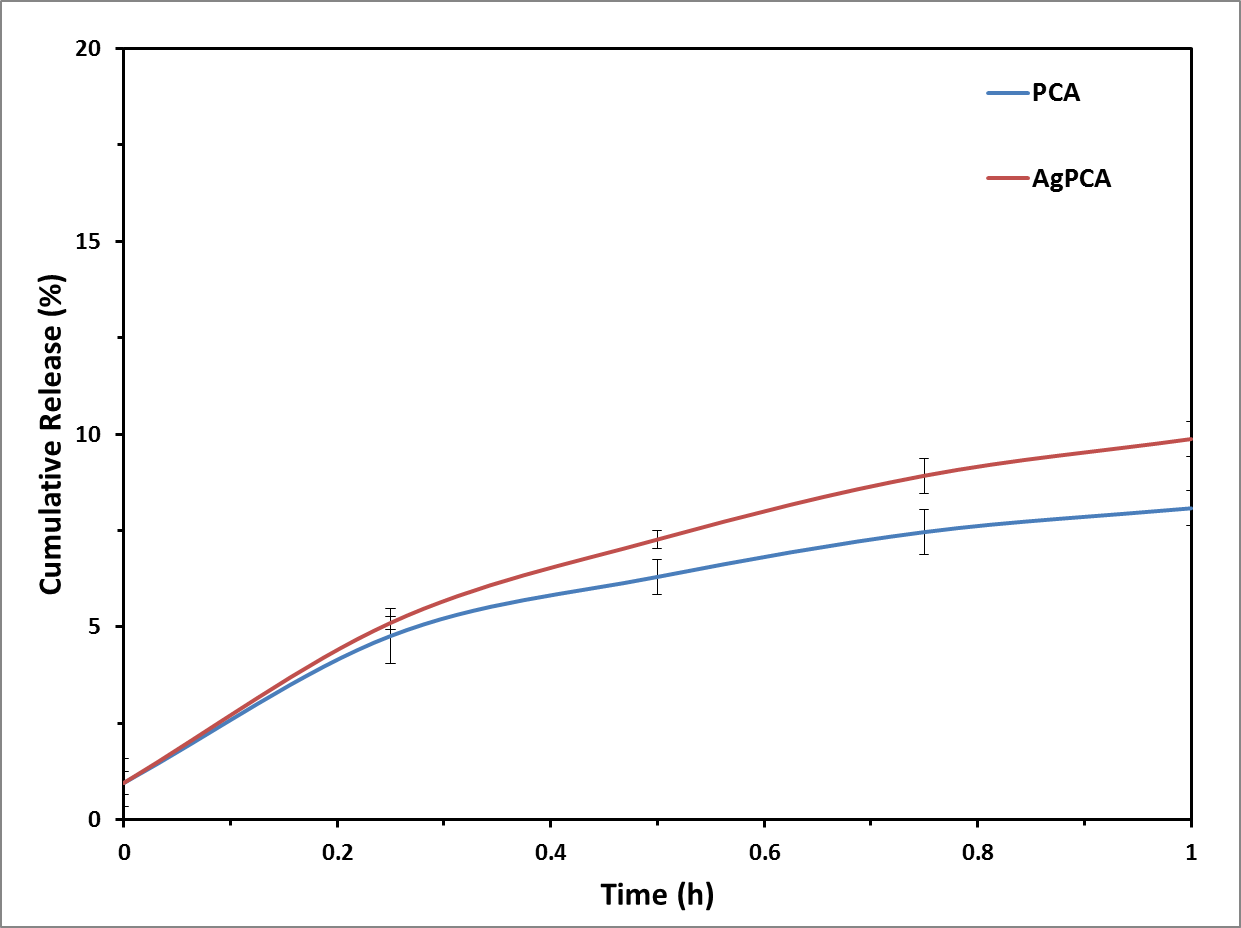

Supplement: Supplementary file 1 [file nanomaterials-09-01423-s001.zip › Supplement/Figure S2.docx]
